# Errors in Figures 3 and 4 and Supplement 1

**DOI:** 10.1001/jamanetworkopen.2026.0081

**Published:** 2026-02-04

In the Original Investigation, “Delayed or Absent First Dose of Measles, Mumps, and Rubella Vaccination,”^1^ published on January 2, 2026, in *JAMA Network Open*, the forest plots in Figures 3 and 4 should be swapped, and in the new Figure 4, the odds ratio (95% CI) under “Fourth month vaccine” for September 2020 to April 2024 should be moved up to be located under the “Fourth month late” vaccines for January 2018 to February 2020. Also, there were formatting errors in a few of the eFigures where some of the labels in the forest plots were cut off. The formatting of these eFigures has been fixed, and the article has been corrected.^1^
